# Asynchrony in terrestrial insect abundance corresponds with species traits

**DOI:** 10.1002/ece3.10910

**Published:** 2024-01-31

**Authors:** Kathryn E. Powell, Tom H. Oliver, Manuela González‐Suárez, Marc S. Botham, Colin A. Harrower, Richard F. Comont, Ian Middlebrook, David B. Roy

**Affiliations:** ^1^ UK Centre for Ecology and Hydrology Wallingford Oxfordshire UK; ^2^ School of Biological Sciences University of Reading Reading UK; ^3^ Bumblebee Conservation Trust London UK; ^4^ Butterfly Conservation Wareham Dorset UK

**Keywords:** asynchrony, Bombus, functional traits, Lepidoptera, stability, trait diversity

## Abstract

Asynchrony in population abundance can buffer the effects of environmental change leading to greater community and ecosystem stability. Both environmental (abiotic) drivers and species functional (biotic) traits can influence population dynamics leading to asynchrony. However, empirical evidence linking dissimilarity in species traits to abundance asynchrony is limited, especially for understudied taxa such as insects. To fill this knowledge gap, we explored the relationship between pairwise species trait dissimilarity and asynchrony in interannual abundance change between pairs of species for 422 moth, butterfly, and bumblebee species in Great Britain. We also explored patterns differentiating traits that we assumed to capture ‘sensitivity to environmental variables’ (such as body mass), and traits that may reflect ‘diversity in exposure’ to environmental conditions and lead to niche partitioning (for example, habitat uses, and intra‐annual emergence periods). As expected, species trait dissimilarity calculated overall and for many individual traits representing response and exposure was positively correlated with asynchrony in all three insect groups. We found that ‘exposure’ traits, especially those relating to the phenology of species, had the strongest relationship with abundance asynchrony from all tested traits. Positive relationships were not simply due to shared evolutionary history leading to similar life‐history strategies: detected effects remained significant for most traits after accounting for phylogenetic relationships within models. Our results provide empirical support that dissimilarity in traits linked to species exposure and sensitivity to the environment could be important for temporal dissimilarity in insect abundance. Hence, we suggest that general trait diversity, but especially diversity in ‘exposure’ traits, could play a significant role in the resilience of insect communities to short‐term environmental perturbations through driving asynchrony between species abundances.

## Graphical abstract

Using data from long‐term monitoring scheme data and novel trait data, we explore the relationship between species trait dissimilarity and their population asynchrony amongst macro‐moths, butterflies and bumblebees. We find that differences in certain traits that correspond with species' exposure to environmental variables, especially phenology‐related traits such as flight period, particularly correlate with population asynchrony between species, giving us further insight into the mechanisms underpinning stability and resilience in insect communities.

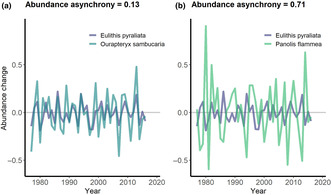

## 1 INTRODUCTION

With biodiversity rapidly changing across the globe, it is important to understand what makes communities more vulnerable to the abiotic anthropogenic environmental drivers widely causing this change, and what leads to greater resilience (Oliver, Heard, et al., 2015). Interspecific asynchrony – i.e., negative temporal correlation between species population sizes (Caruso et al., 2020) – is increasingly recognised to underpin stability, and therefore the resilience, of community and ecosystem‐level dynamics in nature (Craven et al., 2018; Lepš et al., 2018; Valencia et al., 2020; also see Caruso et al., 2020). Asynchronous temporal fluctuations between species can be detectable in time‐series abundance data (Loreau & De Mazancourt, 2008).

Variation in abundance dynamics between species is likely driven by a complex range of interacting biotic and abiotic factors, including genetic drift or stochastic processes, intra‐ and interspecific density dependence, environmental conditions and the functional properties of species and their realised niches (Loreau & De Mazancourt, 2008). These functional properties are referred to as ‘functional traits’. Although there are several definitions across the literature, generally, functional traits consist of measurable morphological, physiological, behavioural, phenological or cultural characteristics which affect how individuals interact with the surrounding environment (Dawson et al., 2021; Luck et al., 2012; Schneider et al., 2019). In general, traits which affect how an individual or species responds to deviations in an environmental pressure such as short‐term climatic perturbations are referred to as ‘functional response traits’, and those that affect how an individual or species impacts the environment around them through a functional role – such as pollination proficiency – are often referred to as ‘functional effect traits’ (Bruelheide et al., 2018; Díaz et al., 2013; Oliver, Heard, et al., 2015). Some complexity arises, seeing as response and effect traits are by no means mutually exclusive – that is to say, there is overlap and correlation between response and effect traits, which can undermine the resilience of function to environmental change (Díaz et al., 2013; Greenwell et al., 2019).

Species with different traits can vary in their sensitivity to certain environmental conditions, leading to variable population‐level responses (Craven et al., 2018; Li et al., 2021; Mumme et al., 2015; van Klink et al., 2019). ‘Dissimilarity’ metrics such as Gower distance between species trait values offer a way of measuring how widely traits vary between species (de Bello et al., 2016). The dissimilarity between traits can result in asynchronous abundance dynamics between species due to differences in their responses to environmental conditions (Craven et al., 2018; Li et al., 2021; Mumme et al., 2015; van Klink et al., 2019). For example, physiological differences associated with variation in body size can lead to divergence in thermal tolerances and, thus, in response to climatic fluctuations (Stevenson, 1985). Species with larger body sizes generally lose heat less quickly which allows them to tolerate a cold snap and maintain or increase their abundance, whilst smaller species tend to be more vulnerable to cold snaps and decrease in abundance (Stevenson, 1985; Verdú et al., 2006). Thus, differences in body size may result in temporal abundance asynchrony between species if, for example, weather conditions favour one species' reproduction and survival over another's.

Although often excluded in the definition of a true ‘response trait’, some of these traits may also be linked to either spatial or temporal niche partitioning between different species, meaning that species vary in their exploitation of resources, as well as exposure to an environmental driver of abundance change in space or time (MacArthur, 1958; Turnbull et al., 2013). For example, considering mobility, a species which can disperse over a wider range may be able to escape the effects of drivers such as localised extreme climate conditions − for example, drought − as opposed to a smaller and less mobile species (Gámez‐Virués et al., 2015). Two species, each with different habitats, can also be subject to fairly extreme differences in microclimatic pressures at any one point in time due to the influence of habitat structure on microclimate (Suggitt et al., 2011); therefore, although their physiological sensitivity to an environmental extreme may not vary per se, species abundances may cycle asynchronously due to the spatial partitioning in their exposure to these variables (Turnbull et al., 2013). Similarly, different species can have different emergence times and lengths of life stages, or their breeding patterns may be exposed to temporal variation in the environment, potentially leading to annual differences in abundance change if one species is subject to worse environmental conditions than the other (Usinowicz et al., 2012). These traits, which can determine the spatial and/or temporal niche partitioning between species, and therefore their exposure to the environment, are not typically thought of as pure ‘functional traits’ due to the lack of measurability from a single individual and the determination of sensitivity to a level of one variable (Dawson et al., 2021). They instead reflect − what we will refer to here as – ‘diversity in exposure’. Nevertheless, they may be inherently important for mediating temporal asynchrony and community stability through their variable effects on species abundance. Dawson et al. (2021) considers the broadening of the definition of functional traits to incorporate aspects of organisms such as diversity in exposure traits, due to the idea that most traits are to some degree ‘functional’ due to their association with the interaction between individuals and/or species and their environment.

Here, we explore the association of species trait dissimilarity with abundance asynchrony across a range of terrestrial insect taxa in Great Britain. Insects play important functional roles in ecosystems, and have found to be declining in diversity, abundance, and biomass in several studies, although there is debate on the extent to which this constitutes a general pattern (Wagner, 2020). At the same time, insects are generally understudied taxa when it comes to understanding the role of traits, and trait‐based mechanisms in ecology; and most understanding of how traits relate to the environment come from studies of plants (Brousseau et al., 2018; Noriega et al., 2018).

We analyse asynchrony in abundance and species trait dissimilarity from pairwise species relationships using long‐term citizen science and standard abundance monitoring datasets in combination with recently published novel l trait data for 422 species of macro‐moths, butterflies, and bumblebees. We explore the relative role of individual traits in contributing to abundance asynchrony. We compare traits deemed to be: (1) ‘sensitivity traits’ that determine species sensitivities to the environment through a link to a direct physiological response to a perturbation, and are therefore functional response traits (in our study: minimum and maximum forewing lengths, estimated dry mass, voltinism and diet breadth and specialisation); as well as (2) ‘exposure traits’: traits in the broader sense which come under the definition given by Dawson et al. (2021) (hostplant and dietary specialisation, habitat use, nesting habits, emergence periods, overwintering stages, and voltinism; some traits could be classified as both categories). Dissimilarity in the exploitation of food plants, nesting quarters and habitat use represents spatial niche partitioning, whilst voltinism, overwintering stage and emergence period represent temporal niche partitioning and so they may determine varying responses amongst species to environmental perturbations. Therefore, we hypothesised that the more these traits differed amongst species, the greater the annual abundance asynchrony between species would be (i.e., a positive correlation between species trait dissimilarity and abundance asynchrony). Through this study we aim to shed light on possible underlying mechanisms that drive asynchrony between species populations and hence contribute to more stable functional communities in terrestrial insects.

## 2 METHODS

### 2.1 Abundance dynamics

We used three different insect abundance time series datasets taken from standardised monitoring projects to calculate relative abundance changes from year‐to‐year for three main insect taxa in Great Britain: (i) macro‐moths, using the ‘Rothamsted Insect Survey’ light trap data (RIS; Woiwod & Harrington, 1994); (ii) butterflies, using the ‘UK Butterfly Monitoring Scheme’ (UKBMS; Botham et al., 2020); and (iii) bumblebees, using the Bumblebee Conservation Trust's BeeWalk survey (Comont & Dickinson, 2020). For each dataset, the ‘Generalised Abundance Index (GAI)’ approach was used to calculate ‘collated abundance indices’ that account for missing count data and the variability in seasonal patterns for species in the three taxonomic groups (Dennis et al., 2013). These indices are an estimate of the expected number of individuals observed on a standardised transect walk (BeeWalk and UKBMS) or trapping event (RIS) during that year. We used collated indices for macro‐moths and butterflies reported by the NERC Environment Information Data Centre. To align methodology for predicting bumblebee annual abundance indices with the GAI methods used for the UKBMS and RIS data, we used the ‘rbms’ package in R to fit the required GAI models to each bumblebee species recorded with a sufficient number of observations from the BeeWalk data (Schmucki et al., 2022). We then calculated relative interannual abundance change values for each species by subtracting the collated annual abundance index for each year from the following year's value, creating a dataset of the annual changes in each species standardised log abundance, following the methodology for calculating relative interannual abundance change outlined in Greenwell et al. (2019).

**FIGURE 1:**
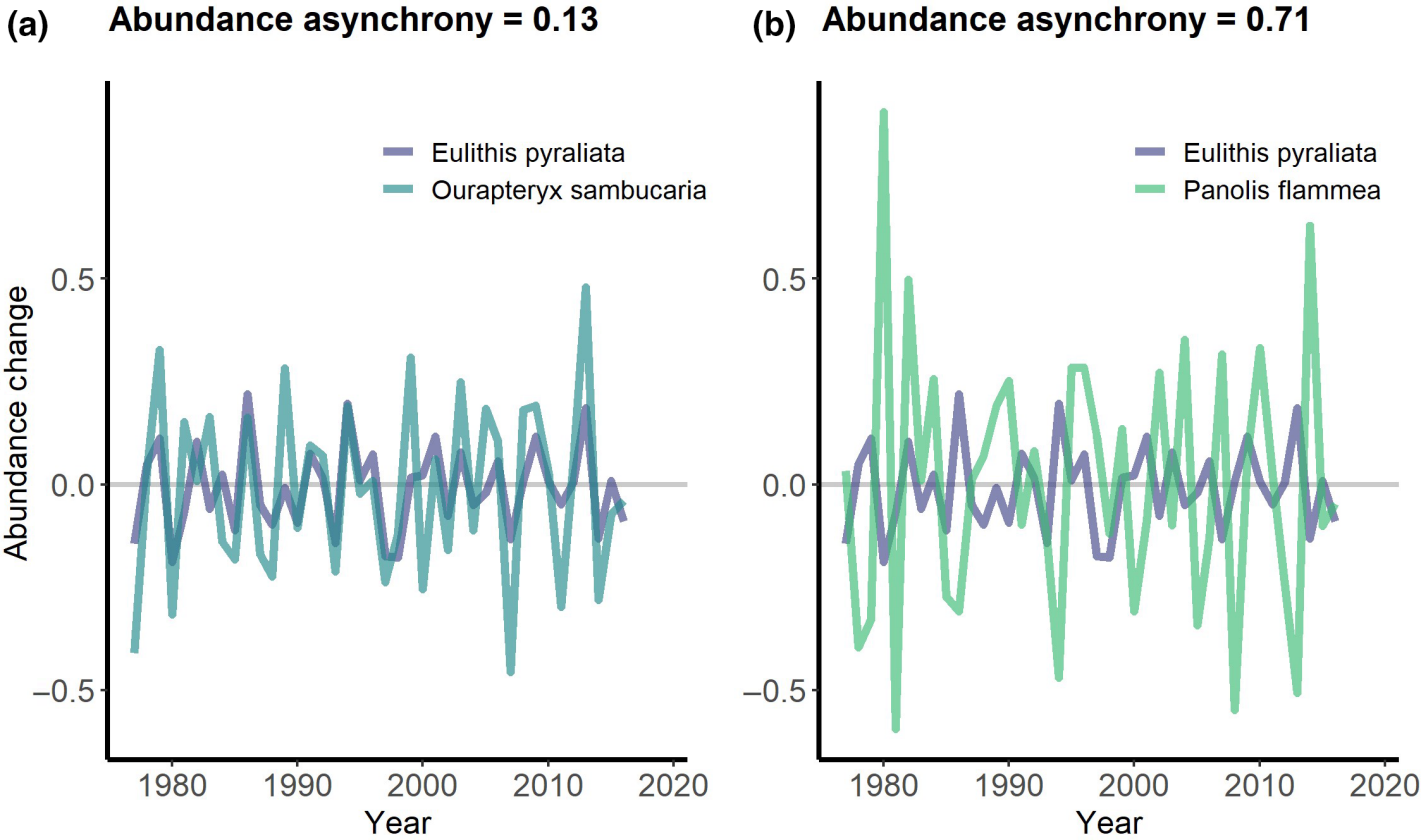
Examples of different levels of asynchrony between interannual abundance dynamics of two pairs of macro‐moth species; (a) *Eulithis pyraliata* (barred straw moth) and *Ourapteryx sambucaria* (swallow‐tailed moth); and (b) *E. pyraliata* and *Panos flammea* (pine beauty moth). In example (a), the high correlation between the species interannual abundance change values results in a calculated abundance asynchrony value of 0.13 (where asynchrony lies between 0 and 1, with 0 being completely synchronous and 1 being completely asynchronous); in example (b), the two species cycle more asynchronously in abundance, with lower correlation between their abundance dynamics, resulting in a calculated asynchrony value which is closer to 1 (0.71).

We then examined interannual abundance dynamic asynchrony between pairs of species using distance matrices. Pairwise distance values were calculated to produce the matrix ***M*** such that:

$$M=\left(1-K\right)/2$$

where ***K*** is a matrix of pairwise Pearson's correlation coefficients calculated from inter‐annual abundance change values using the corr() function in R (version 4.0.3). This intentionally produced values between 1 (completely asynchronous) and 0 (completely synchronous) so that they were on a comparable scale to trait dissimilarity values (see below). See Figure 1 for an example of different levels of asynchrony between species from the RIS dataset. We produced matrices for butterfly, moth and bumblebee abundance. It should be noted that in the BeeWalk dataset, species recorded as *Bombus quorum*, *Bombus magnus*, *Bombus cryptarum* and *Bombus terrestris* had to be combined into one species complex (which we call *Bombus lucorum*/*terrestris*), due to taxonomic identification difficulties of the worker castes in the field (Carolan et al., 2012).

### 2.2 Traits

We collated and compiled functional trait data for all species using guidebooks for bumblebee species (Falk, 2015) and a compiled Lepidoptera trait dataset for macro‐moths and butterflies (Cook et al., 2021). We focused on traits reported in the literature as showing correlation with abundance, distribution, or extinction risk in response to environmental drivers for any of the three taxa, which we considered to be appropriate traits under the definition outlined in the introduction. We acknowledge that some traits included – such as habitat – may not fall under the definition of a trait in other research because an organisms' habitat is the result of an interaction between traits and environmental conditions (e.g., the availability of a habitat). However, for the purpose of this study, habitat use was retained as an ‘exposure’ trait that could influence asynchrony caused by exposure to habitat‐specific environmental stressors, such that species found in different habitats will be exposed to different conditions. A full list of traits used in the analysis are outlined in Table 1. For bumblebees, as we collated these data ourselves, we coded traits in line with the values used in the published Lepidopteran data to keep this as standardised as possible, i.e., trait values were mostly categorical and coded in binary (1 or 0, depending on presence of trait in species) with multiple columns for each trait category that represent the ‘dimensions’ (or indeed ‘levels’ – or different aspects) of a trait (e.g., monophagous, oligophagous, or polyphagous categories for the Hostplant or diet Specialism trait; species can be coded as ‘1’ for multiple columns within traits if traits were not mutually exclusive, e.g., for Hostplant Category, when multiple types of hostplant are used by the species), unless the trait was a continuous variable such as body mass estimates. As eusocial bumblebee species castes vary widely in their body size and forewing lengths, e.g., between queens and workers, size and mass measurements relating to queens was used in trait matrices to be comparable with cuckoo bumblebee species which do not have workers. We assumed this would not change directions of relationships between species in our analyses as bumblebee species with larger queens have larger workers, and vice versa.

**TABLE 1:** List of traits used for building species trait dissimilarity matrices.

Trait group	Trait abbreviation	Description	Functional association	Combined traits dissimilarity weighting	Relevant taxa		
					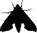	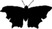	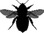
Nesting habits	Pupal nesting habit (PH)	Location for the pupal and/or nesting stages (underground, soil surface, on hostplant, within hostplant, on other vegetation, within stone walls, and dead wood)	Links to extinction risk and distribution; exposure to different microhabitats, microclimates, and disturbance (exposure)	0.167	✓	✓	✓
Body size	Forewing minimum (FMI)	Minimum forewing length (mm)	Predictor of dispersal and population declines, linked to physiological sensitivity (sensitivity)	0.0556	✓	✓	
Body size	Forewing maximum (FMA)	Maximum forewing length (mm)	Predictor of dispersal and population declines, linked to physiological sensitivity (sensitivity)	0.0556	✓	✓	✓
Body size	Estimated body mass (EDM)	Dry mass estimate (mg), calculated using models from Kinsella et al. (2020) for moths and Cane (1987) for bumblebees	Predictor of dispersal and population declines, linked to physiological sensitivity (sensitivity)	0.0556	✓		✓
Phenology	Voltinism (V)	Number of broods per year (univoltine: 1 brood; multivoltine: 2 or more broods)	Links to recovery from disturbance, exposure to different drivers of change temporally (sensitivity and exposure)	0.0278	✓	✓	✓
Phenology	Egg stage (ES)	Months during which individuals are eggs (Jan–Dec)	Associated with extinction risk and distribution, exposure to different drivers of change temporally (exposure)	0.0278	✓	✓	
Phenology	Pupal stage (PS)	Months during which individuals are pupae (Jan–Dec)	Associated with extinction risk and distribution, exposure to different drivers of change temporally (exposure)	0.0278	✓	✓	
Phenology	Larval stage (LS)	Months during which individuals are larvae (Jan–Dec)	Associated with extinction risk and distribution, exposure to different drivers of change temporally (exposure)	0.0278	✓	✓	
Phenology	Adult stage (AS)	Months during which individuals are adults and actively flying (Jan–Dec)	Associated with extinction risk and distribution, exposure to different drivers of change temporally (exposure)	0.0278	✓	✓	✓
Phenology	Overwinter stage (OS)	The life stage that species overwinter in (egg, larva, pupa or adult)	Correlated with both distribution, extinction risk and abundance trend, exposure to different drivers of change temporally (exposure)	0.0278	✓	✓	✓
Diet	Hostplant number (HN)	The number of host plant species used by larvae	Correlated with dispersal and range size (exposure)	0.0556	✓	✓	
Diet	Hostplant (or diet) specificity (HS)	Whether species are monophagous, oligophagous, or polyphagous	Correlated with dispersal and range size (exposure)	0.0556	✓	✓	✓
Diet	Hostplant category (HC)	Plant category that host‐plants come under (e.g., trees, grasses, sedges, mosses, etc.)	Correlated with both increases and decreases in abundance trends; exposure to different drivers of change spatially and temporally (exposure)	0.0556	✓	✓	
Habitat	Habitat USE (H)	Typical habitat (woodland, heathland, moorland, grassland, wetland, coastal, montane, urban/agricultural)	Preference and specificity can predict population declines; exposure to different drivers of change spatially and temporally (exposure)	0.167	✓	✓	✓

We used the gawdis() function of the ‘Gawdis’ package in R (de Bello et al., 2020) to calculate Gower distances for trait values between each pair of species across butterflies, macro‐moths and bumblebees. We constructed trait distance matrices for each individual trait, as well as a ‘combined traits’ distance matrix for each taxonomic group and combination of groups. For this combined traits matrix, as there was correlation between a number of traits which described similar aspects of species ecologies, as well as the challenge of addressing and combining both categorical and continuous traits, we used the grouping argument of the gawdis() function to combine traits that we considered to come together to describe one functional aspect (a ‘trait group’). This methodology alters the weighting of each individual ‘raw’ trait within trait groups so that each final trait group contributes equally to the resulting final combined traits dissimilarity matrix, in line with methods described in (de Bello et al., 2020). Using this function to combine and condense traits allowed us to reduce bias given to certain traits that were closely correlated to others in the dataset, whilst avoiding removing traits from the analysis, which may have otherwise reduced the accuracy of the Gower distances calculated between species pairs. The grouping argument identified five trait groups which can broadly be described as: nesting habits, body size, phenology, diet, and habitat (Table 1).

### 2.3 Phylogenetic distance

Phylogenetically related species are more likely to show similar trait values due to their shared evolutionary history, thus, trait distance between species may be phylogenetically patterned. We wanted to test, therefore, for evidence of phylogenetic signal in pairwise trait distances, and to examine whether temporal asynchrony of abundance dynamics could still be explained by trait distances regardless of phylogenetic constraints. We use the Lepidopteran phylogenetic tree in Pöyry et al. (2017, Appendix S1) for macro‐moths and butterflies, and the bumblebee phylogenetic tree from Cameron et al. (2007). We then calculated distance matrices between each pair of species within taxonomic groups from the branch lengths of the phylogenies using the cophenetic.phylo() function in the ‘ape’ package in R (Paradis & Schliep, 2019). For bumblebee species we calculated average branch lengths between each species and the *B. lucorum*/*terrestris* species complex by taking the mean of the branch lengths between each species and *B. cryptarum*, *B. magnus*, *B. lucorum* and *B. terrestris*.

### 2.4 Testing correlative relationships between distance matrices

For each of the following statistical analyses, only species for which we could obtain data for all three datasets (i.e., abundance dynamics, traits and phylogenetics datasets for each taxonomic group), were included in pairwise distance matrices. We created Mantel tests using the Mantel() function with 10,000 permutations in the ‘ecodist’ package in R (Goslee & Urban, 2007) to test whether there was correlation between pairwise temporal abundance asynchrony and species trait dissimilarity matrices. 95% confidence limits and *p* values were constructed using bootstrap resampling for 10,000 repeats. We deemed Mantel *r* values to be significant based on the ‘*p* value 1’ output from Mantel tests which tests the null hypothesis that Mantel *r* < 0, based on a one‐tailed *t*‐test.

Using the same method for Mantel tests above, we tested both species trait dissimilarity and abundance asynchrony matrices in macro‐moths, butterflies, and bumblebees against phylogenetic distance matrices. Seeing as phylogenetic distance was found in all cases to be significantly positively correlated with abundance asynchrony, we also tested the relationship between trait distance and abundance asynchronies once the correlation between phylogeny and abundance was removed, i.e., accounting for phylogenetic differences between species, through partial Mantel tests. This allowed us to explore whether traits alone could explain abundance asynchrony beyond the effects of phylogenetics.

Partial tests to estimate the similarity between trait distance and abundance asynchrony were structured as follows:

Abundance asynchrony~species trait dissimilarity+phylogenetic distance

where each of the three elements is a distance matrix constructed according to the methodology outlined above.

## 3 RESULTS

Sample sizes in terms of numbers of species for each pairwise asynchrony, species trait dissimilarity and phylogenetic distance matrix construction were as follows: macro‐moths, *n* = 358 species; butterflies, *n* = 48; and bumblebees, *n* = 16.

### 3.1 Relationship between traits and abundance dynamics

#### 3.1.1 Overall functional dissimilarity: Combined traits analyses

**FIGURE 2:**
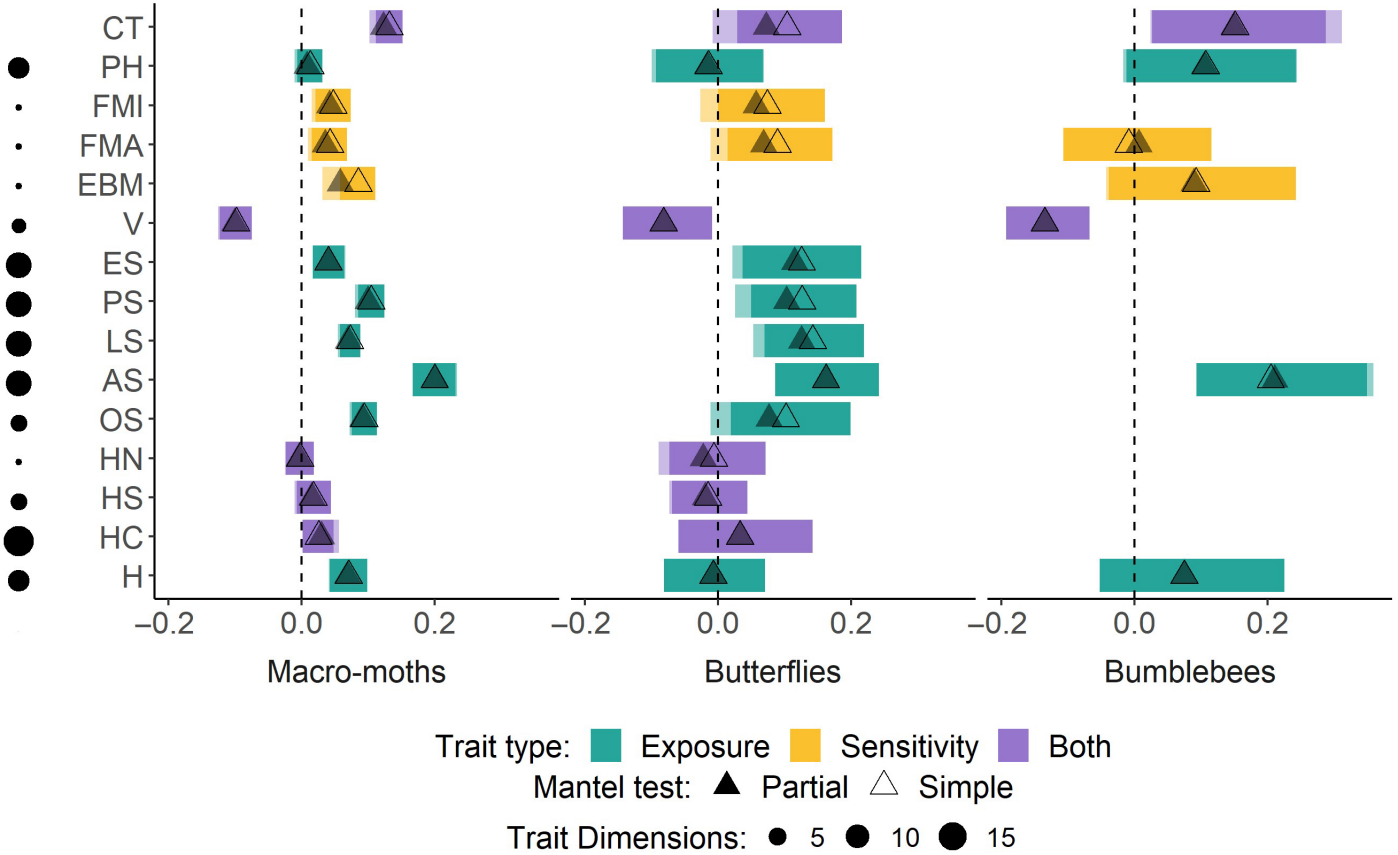
Mantel *R* values extracted from both simple Mantel tests (investigating the correlation between matrices of pairwise species trait dissimilarity and abundance asynchrony) and partial Mantel tests (accounting for phylogenetic distance between species) for macro‐moths (*n* = 358), butterflies (*n* = 48), and bumblebees (*n* = 16). The initials on the y axis refer to the combined traits and the individual traits tested from Table 1, alongside a visual representation of the number of dimensions used to code each individual trait within trait databases, and are ordered from top to bottom as the following: AS, adult stage (*n*, 12); CT, combined traits; EBM, estimated body mass (*n*, 1); ES, egg stage (*n*, 12); FMA, forewing maximum (*n*, 1); FMI, forewing minimum (*n*, 1); H, habitat (*n*, 8); HC, hostplant category (*n*, 18); HN, hostplant number (*n*, 1); HS, hostplant (or diet) specificity (*n*, 4); LS, larval stage (*n*, 12); OS, overwintering stage (*n*, 4); PH, pupal nesting habit (*n* dimensions [hereon referred to as ‘*n*’], 8); PS, pupal stage (*n*, 12); V, voltinism (*n*, 3).

In all three species groups, species trait dissimilarity was significantly positively correlated with abundance asynchrony (Moths: Mantel *r* = .13, *p* < .001, *n* = 358; Butterflies: Mantel *r* = .103, *p* < .05, *n* = 48; and Bumblebees: Mantel *r* = .15, *p* < .05, *n* = 16; Figure 2). Hence, when two species of moth, butterfly or bumblebee are less similar in terms of their overall trait profiles, then those two species will tend to have divergent abundance changes.

When accounting for the correlation between phylogenetic distance and abundance asynchrony using partial Mantel tests, this relationship between trait distance and abundance asynchrony remained significant in all groups except butterflies (partial Mantel; Macro‐moths: Mantel *r* = .12, *p* < .001, *n* = 358; Butterflies: Mantel *r* = .07, *p* = .12, *n* = 48; and Bumblebees: *r* = .15, *p* < .05, *n* = 16; Figure 2).

#### 3.1.2 Separate trait analyses

We found variation in the association between individual functional response traits and abundance asynchrony across species groups.

For macro‐moths, dissimilarity in habitat use, life stage periods (egg, larval, pupal, and adult), overwintering stage, minimum and maximum forewing lengths, estimated body mass were all significantly positively correlated with abundance asynchrony (both simple and partial Mantel, Figure 2; partial Mantel presented in Appendix S2). However, number, type, and specificity of hostplants; and pupal habit were not significantly correlated with abundance asynchrony, whilst voltinism was negatively correlated with abundance asynchrony (both simple and partial Mantel, Figure 2; partial Mantel presented in Appendix S2).

For butterflies only dissimilarity in the life stage periods remained positively significant when the association between phylogenetic relatedness and abundance asynchrony was accounted for in partial Mantel tests (Figure 2, Appendix S2). For simple Mantel tests, dissimilarity in overwintering stage and maximum forewing lengths were also marginally positively associated with abundance asynchrony amongst butterflies, their credible intervals overlapping with zero once phylogenetic patterns were included in models (Figure 2).

For bumblebees, data for fewer traits were available. Adult period (or flight window) dissimilarity was significantly positively correlated with abundance asynchrony, while voltinism dissimilarity was significantly negatively correlated (Figure 2, Appendix S2). Other explored traits (forewing length, pupal (nesting) habit, estimated body mass and habitat use) were not significantly correlated with abundance asynchrony (Figure 2, Appendix S2).

## 4 DISCUSSION

In line with our hypothesis, we found species trait dissimilarity to be generally positively associated with abundance asynchrony across different species of macro‐moths, butterflies, and bumblebees (i.e., species with different traits tend to have asynchronous population dynamics). Phylogenetic distance was also positively associated with abundance asynchrony, possibly because there is phylogenetic signal amongst traits that we tested and/or additional traits that we did not include that have an influence on asynchrony (Díaz et al., 2013). However, our traits could still explain a significant amount of abundance asynchrony even when taking phylogenetic relationships into account (Figure 2).

There was variation across the relationships between traits and abundance dynamics for different taxonomic groups, and for different traits. For example, traits such as habitat use and annual timings of life history stages were the most strongly associated with asynchrony, with adult stage consistently having the strongest association across all groups (although for bumblebees, fewer traits were able to be tested due to data gaps). This suggests species with similar adult emergence have more similar population dynamics than those emerging in different months, which might be expected as they are exposed to more similar weather conditions (e.g., a spring drought could affect all species in early larval stages similarly) (Zhang, Bao, et al., 2022). Intra‐annual variation in environmental factors, such as seasonal weather change in the UK, mean that species emerging at different times will vary in their exposure to these factors (Zhang, Hautier, et al., 2022). Intra‐annual asynchrony has been found to increase intra‐annual stability and support co‐existence of species in plant communities (Usinowicz et al., 2012; Zhang, Bao, et al., 2022); our results suggest that such factors may be important for asynchrony in insect communities, and for dissimilarity in inter‐annual abundance dynamics between species. Drivers of phenological shifts and intra‐annual abundance dynamics of these insect taxa, such as climate change (Davies, 2019; McCauley & Mabry, 2011; Stemkovski et al., 2020; Visser & Holleman, 2001; Wuethrich, 2000), may result in greater overlap in adult emergence as springs generally become warmer and species that usually emerge in late spring or early summer begin to emerge earlier in the year (O'Neill et al., 2012). Given our results, there is a possible risk that such phenological shift and overlap may decrease inter‐annual asynchrony between species, although this effect could also potentially be offset by increasing the number of broods per year during warmer years where there is a longer time period of reproduction (Altermatt, 2009; Zografou et al., 2021).

Dissimilarity in species habitat use was also significantly correlated with abundance asynchrony in macro‐moths. This trait was not significant for butterflies or bumblebees in our study; however, this result may be an artefact of limited species pools for these taxa, resulting in a larger proportional overlap in habitat use for the species considered in the study. Differences in habitat use leads to pairs of species being more likely to exploit different resources as well as experience different local environmental conditions – such as microclimatic extremes – and pressures at any one time point (Gilbert et al., 2020; Suggitt et al., 2011; Van Ruijven & Berendse, 2005). We suggest that not only ‘response diversity’ – in the sense that species vary in their sensitivity to environmental pressures – drives asynchronous abundance dynamics, but that dissimilarity in exposure to different environmental drivers over time and space, both through phenological dissimilarity and difference in habitat use, is an important aspect of inter‐annual asynchrony amongst these taxa (Albrecht & Gotelli, 2001).

In contrast, dissimilarity in other traits such as those linked to diet, specialism and pupal habits were generally not correlated with abundance asynchrony. We also found dissimilarity in voltinism to have negative correlation with abundance asynchrony (i.e., species with different voltinism were *more* similar in population dynamics). This may be because, in Lepidoptera for example, the first generations of most multivoltine species tend to coincide with the flight periods of early flying species and the second generation with late flying species, reducing dissimilarity between multivoltine and univoltine species pairs. As our analysis shows, dissimilarity in species adult emergence throughout the year increases population asynchrony, so perhaps this is more important than being univoltine or multivoltine per se. It is expected that having species with more than one generation in a year would increase resilience within a population because a subset will be less exposed to an extreme event that strongly negatively affects a species at a given time (Knell & Thackeray, 2016). Thus, their annual abundance could still be high while the univoltine species emerging at the same time will be low. Our negative result for voltinism is interesting as it suggests this may not be the case for stabilisation of community abundance. To examine this phenomenon from another angle, we may see this pattern because species with one brood are less likely to have them at the same time during the year as other univoltine species, or reach adult stages at different times, and so have differential exposure to environmental perturbations, leading to decoupling of abundance dynamics. Within the macro‐moths, many species fly at times of year when other insects are inactive, in late autumn, winter and early spring (Soszyńska‐Maj, 2015). These are generally univoltine, and are adapted to cooler temperatures (Heinrich, 1987). Univoltine moth species generally show much greater dissimilarity amongst their flight periods and brood period timing than multivoltine species, where species are active across the entire calendar year and overlap. However, this is less likely to be the case for bumblebees and butterflies whose brood timings are more seasonally restricted.

For traits that we deemed to be linked to species sensitivity to the environment, we found body size metrics – including forewing length and estimated dry mass, to be positively associated with abundance asynchrony in macro‐moths. However, although we had no data on estimated body mass for butterflies, the effect for forewing length – which likely strongly associates with body mass – was lower (closer to 0) in butterflies, which corresponds with similar results in Greenwell et al. (2019). We found no effect of forewing length or body mass in bumblebees. Body size is frequently described as a functional response trait, as larger body size is predicted to make species more vulnerable to environmental stressors and increase extinction risk (Coulthard et al., 2019), although this may sometimes be offset by the ability of larger species to generally disperse more quickly to reach more favourable environments (Sekar, 2012). This complexity of how traits result in varying individual and population responses to environmental drivers – over short and long‐term timescales and over different spatial scales – has been noted in recent studies linking moth traits to trends (Tordoff et al., 2022). For example, from the results of our study, it's possible that temperature fluctuations between years favour a particular minimum or maximum body size, resulting in varied species‐level responses and resulting in asynchronous dynamics between species of different size (Mattila et al., 2011).

We found some phylogenetic signal amongst traits across macro‐moth and butterfly taxa, with more phylogenetically distant pairs of species having less similar trait complexes (Appendix S2). This is expected given that traits evolve over time and species diverge genetically and phenotypically as they adapt to new environmental challenges (Díaz et al., 2013). In butterflies, this has been found to extend to the ‘exposure traits’ that we test here such as adult emergence, the date of which shows evidence of local adaption in response to climate (Roy et al., 2015). Hence, as we hypothesised species trait dissimilarity to also be positively associated with population asynchronies between species, we would expect that phylogenetic distance also correlates with abundance asynchrony, which our results supported (Appendix S2). This phylogenetic ‘signal’ may help explain why some traits become decoupled from population asynchronies when accounting for phylogenetic relatedness amongst species, and for most traits shifts the Mantel R value closer to 0.

An important limitation to our analysis is that the features of the datasets we use here only allowed us to explore abundance asynchrony and traits at large scales – spatially, using annual abundance indices at a national level; and taxonomically, using mean values for species traits when there is ample evidence that the traits we use in this analysis can vary intraspecifically (Wong & Carmona, 2021). For example, some traits such as habitat use, or host plant use are likely to be important for species on different spatial scales to those considered in our analysis. Whilst the coarseness of the available data did not allow us to look at this, or further into more nuanced traits – for example, floristic richness and microhabitat use, which may play a more significant role in bumblebee niches (e.g., Scriven et al., 2015) – our results still support a temporal and spatial niche‐partitioning hypothesis for abundance asynchrony within Lepidopteran and bumblebee communities at the wider spatial scale (Isbell et al., 2009).

Although our analysis suggests that there are positive associations between species trait dissimilarity, phylogenetic distance and abundance asynchrony, several factors may have reduced the power of our results in explaining the role of traits in abundance asynchrony and may help explain some of the variation between different taxonomic groups. For each dataset, the length of population time series varied. Additional analyses found that there was a significant positive effect of time series length on the strength of the correlation between species trait dissimilarity and abundance asynchrony in butterflies and macro‐moths, as well as the likelihood of detecting significance in this relationship (Appendix S1). The number of species included in each dataset and for each taxonomic group also varies markedly. One of the reasons that lower species numbers may result in a lack of signal between traits and asynchrony may be that the variability in traits amongst species within these taxa may be lower. We explored the spread of the species trait dissimilarity data however and found a normal distribution across similar ranges of species trait dissimilarity in the standardised matrices used for analyses (Appendix S3). For bumblebees, however, there was a right‐hand skew in the spread of abundance asynchrony values, with more pairs having lower asynchrony; this may go some way to explain a lack of correlation between traits and asynchrony in this group. Testing the effect of increasing the number of species included in the analysis did not change the overall average correlation between trait distance and abundance asynchrony, although using low numbers of species (<50) resulted in large variation in the results as well as high confidence intervals which lead to less chance of a signification correlation. Although we could only investigate this issue in Lepidopteran time series, we suggest that it may generally explain why we found fewer butterfly traits and only one bumblebee trait to be significant.

Our findings that dissimilarity in traits – both when traits are associated with sensitivity to environmental variables and when traits are associated with spatial and temporal niche partitioning – are linked to interspecific asynchrony in insect communities, could have wider implications for understanding the functional mechanisms behind long‐term stability and resilience (Oliver, Heard, et al., 2015). As asynchrony is frequently found to be one of the most important drivers of stability within biological communities, our results support the view that communities with diverse traits can maintain functional resilience to environmental perturbations (Craven et al., 2018; Loreau & de Mazancourt, 2013; Sasaki et al., 2019). Beyond our understanding that asynchrony can emerge from dissimilarity in pure ‘functional response trait’ values and *sensitivity* to environmental drivers, we show that traits relating to aspects of species and their spatial and temporal niche partitioning (i.e., their *exposure* to particular environmental conditions) may be important for mediating between‐year asynchrony (Dawson et al., 2021). This may be important in the context of future environmental change, given these ‘traits’, such as flight period, are usually more plastic; for example, shifting phenology with climate change may result in species advancing their emergence times to earlier in the year and decreasing dissimilarity in their flight periods, with potential negative effects on between‐year asynchrony between species (Stewart et al., 2020).

A greater understanding of the relationship between both perturbations and long‐term environmental change, intra‐annual abundance dynamics and inter‐annual asynchrony in these species may help us further understand how resilient communities are under anthropogenic drivers such as climate and land use change (Bellard et al., 2012; He et al., 2019; Oliver, Isaac, et al., 2015).

## AUTHOR CONTRIBUTIONS

**Kathryn E. Powell:** Conceptualization (lead); data curation (equal); formal analysis (lead); investigation (lead); methodology (lead); project administration (equal); visualization (lead); writing – original draft (lead). **Tom H. Oliver:** Conceptualization (supporting); funding acquisition (supporting); methodology (supporting); project administration (supporting); supervision (supporting); validation (supporting); writing – review and editing (equal). **Manuela González‐Suárez:** Supervision (equal); validation (equal); visualization (supporting); writing – review and editing (equal). **Marc S. Botham:** Data curation (equal); validation (supporting); writing – review and editing (equal). **Colin A. Harrower:** Data curation (equal); writing – review and editing (equal). **Richard F. Comont:** Data curation (equal); writing – review and editing (equal). **Ian Middlebrook:** Data curation (equal). **David B. Roy:** Funding acquisition (lead); project administration (equal); resources (lead); supervision (lead); writing – review and editing (equal).

## CONFLICT OF INTEREST STATEMENT

The authors declare no conflicts of interest to their knowledge.

## Supporting information


Appendix S1.

Appendix S2.

Appendix S3.

## Data Availability

The data analysed in this paper come from a variety of sources. The abundance data for insects can be accessed as follows: UK Butterfly Monitoring Scheme annual abundance index data are available via the NERC Environment Information Data Centre portal (EIDC; https://catalogue.ceh.ac.uk/documents/571a676f‐6c32‐489b‐b7ec‐18dcc617a9f1); Rothamsted Insect Survey macro‐moth abundance data from light traps can be accessed via the Rothamsted Research webpage (https://insectsurvey.com/moth‐data); and the Bumblebee Conservation Trust BeeWalk data can be accessed through the online data‐sharing platform ‘Figshare’ (https://figshare.com/articles/dataset/BeeWalk_dataset_2008‐19/12280547). Trait data for butterflies and macro‐moths is accessible via the EIDC (https://catalogue.ceh.ac.uk/documents/5b5a13b6‐2304‐47e3‐9c9d‐35237d1232c6), and the traits for bumblebees collated by the authors of this paper from sources such as Falk (2015) have been deposited with the EIDC (https://eidc.ac.uk/). Phylogenetic data were accessed by contact with the relevant authors of the published material. The final abundance, phylogenetic and trait distance matrices used for the main analyses are accessible via github (https://github.com/katpow/asynchrony‐traits).
