# Supplementary material for: Asynchrony in terrestrial insect abundance corresponds with species traits
Source: Ecol Evol. 2024 Jan 31;14(2):e10910. doi: 10.1002/ece3.10910 (PMC10830349; doi:10.1002/ece3.10910)
Supplement: Supplementary file 1 — Appendix S1. [file ECE3-14-e10910-s001.docx]

Appendix S1

**The role of time series length and matrix size in the sign and strength of relationship between trait distance and abundance asynchrony**

As the population time series were of different lengths depending on datasets, and our Mantel tests were carried out on matrices of varying sizes, we wanted to test the effect of both time series length and matrix size on the outcome of mantel tests, in terms of the strength of the correlation between matrices (mantel r) and the detection of significant values (p values). To do this, we resampled the Lepidopteran (macro-moth and butterfly) population time series 1000 times using randomised start and end years, thus allowing us to create 1000 different matrices of pairwise population asynchrony values for all 470 Lepidopteran species, using time series of varying lengths (between 5 and 42 years). We then carried out mantel tests between each of these population matrices and the trait and phylogenetic matrices. We also resampled Lepidopteran time series another 1000 times, using the full time series length (42 years) but included different numbers of species using randomized species selection in the final matrices (between 5 and 470 species). Mantel tests were then carried out between each of these matrices and the trait / phylogenetic matrices (which were trimmed down to the relevant species included from time series), as well as to test for phylogenetic signal in traits when matrices were of different size. We used generalized linear models with a binomial error to test the effects of time series length and matrix size on the outcome of mantel tests (mantel r value and p values 1,2, and 3). Resampling the macro-moth and butterfly time series data revealed there to be a significant positive effect of time series length on the mantel r values produced when testing for multi-trait and phylogenetic signal in population dynamics, as well as on the detection of significance. We found no effect of matrix size in general on the mantel r value, but significant effects on the p values produced in mantel tests, for all three comparisons between population asynchrony, functional trait dissimilarity and phylogenetic distance.


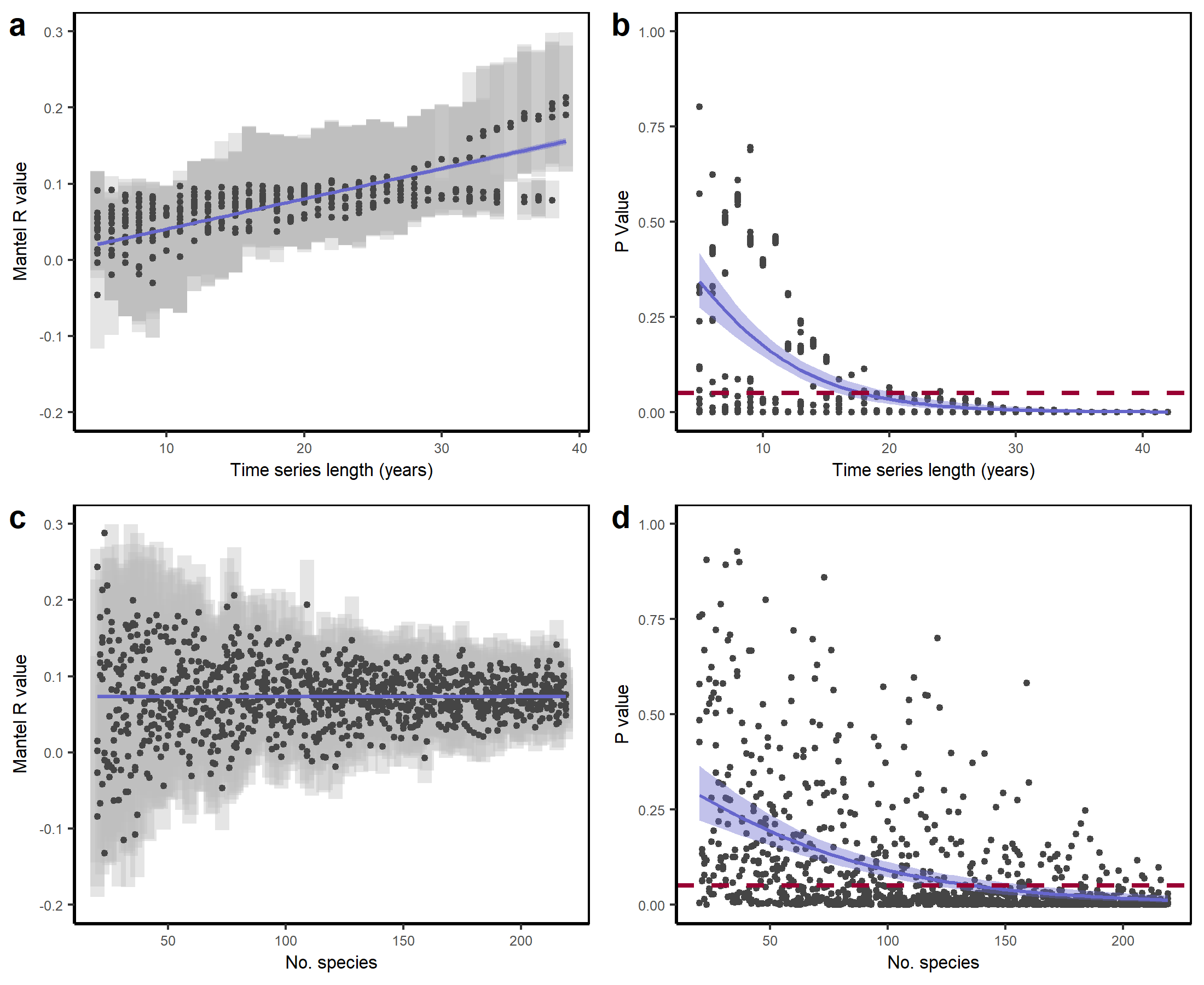


**Fig S1.** The outcome of mantel tests comparing population asynchrony with functional trait dissimilarity in Lepidoptera when resampled 1000 times. Panels a) and b) show the mantel r values and P values for tests conducted in communities resampled with increasing length of time series data respectively. Panels c) and d) show the mantel r values and p values for tests conducted in communities resampled with an increasing number of species included in the analysis.
