# Supplementary material for: Asynchrony in terrestrial insect abundance corresponds with species traits
Source: Ecol Evol. 2024 Jan 31;14(2):e10910. doi: 10.1002/ece3.10910 (PMC10830349; doi:10.1002/ece3.10910)
Supplement: Supplementary file 2 — Appendix S2. [file ECE3-14-e10910-s002.docx]

Appendix S2

**Table S2.** Mantel r values calculated for all partial mantel tests conducted between abundance asynchrony and trait dissimilarity matrices

| Taxa | Matrix_2 | Mantel R | Lower CI (95%) | Upper CI (95%) | P value |
| --- | --- | --- | --- | --- | --- |
| Macro-moths | All traits | 0.123 | 0.102 | 0.143 | 0.00 |
|  | Adult Stage | 0.201 | 0.168 | 0.234 | 0.00 |
|  | Pupal Stage | 0.100 | 0.080 | 0.119 | 0.00 |
|  | Overwintering Stage | 0.091 | 0.072 | 0.111 | 0.00 |
|  | Larval Stage | 0.070 | 0.055 | 0.086 | 0.00 |
|  | Habitat | 0.069 | 0.041 | 0.097 | 0.00 |
|  | Estimated Body Mass | 0.058 | 0.031 | 0.083 | 0.00 |
|  | Forewing Minimum | 0.041 | 0.015 | 0.067 | 0.03 |
|  | Egg Stage | 0.041 | 0.017 | 0.065 | 0.03 |
|  | Forewing Maximum | 0.035 | 0.009 | 0.061 | 0.05 |
|  | Hostplant Category | 0.029 | 0.002 | 0.056 | 0.10 |
|  | Photoperiod | 0.027 | -0.004 | 0.059 | 0.16 |
|  | Hostplant Specificity | 0.015 | -0.010 | 0.042 | 0.23 |
|  | Pupal Habit | 0.008 | -0.011 | 0.027 | 0.31 |
|  | Hostplant Number | -0.004 | -0.025 | 0.017 | 0.58 |
|  | Voltinism | -0.101 | -0.126 | -0.077 | 1.00 |
| Butterflies | All traits | 0.072 | -0.008 | 0.166 | 0.13 |
|  | Adult Stage | 0.162 | 0.087 | 0.242 | 0.00 |
|  | Larval Stage | 0.125 | 0.053 | 0.203 | 0.00 |
|  | Egg Stage | 0.115 | 0.022 | 0.206 | 0.04 |
|  | Pupal Stage | 0.103 | 0.026 | 0.187 | 0.03 |
|  | Overwintering Stage | 0.076 | -0.011 | 0.178 | 0.08 |
|  | Forewing Maximum | 0.068 | -0.012 | 0.160 | 0.12 |
|  | Forewing Minimum | 0.057 | -0.027 | 0.148 | 0.17 |
|  | Hostplant Category | 0.033 | -0.054 | 0.140 | 0.34 |
|  | Habitat | -0.006 | -0.079 | 0.071 | 0.51 |
|  | Pupal Habit | -0.016 | -0.100 | 0.069 | 0.60 |
|  | Hostplant Specificity | -0.020 | -0.074 | 0.042 | 0.67 |
|  | Hostplant Number | -0.023 | -0.089 | 0.054 | 0.62 |
|  | Voltinism | -0.082 | -0.142 | -0.011 | 0.91 |
| Bumblebees | All traits | 0.149 | 0.024 | 0.312 | 0.07 |
|  | Adult Stage | 0.211 | 0.094 | 0.359 | 0.03 |
|  | Pupal Habit | 0.105 | -0.017 | 0.244 | 0.19 |
|  | Estimated Body Mass | 0.089 | -0.043 | 0.235 | 0.24 |
|  | Habitat | 0.074 | -0.050 | 0.226 | 0.25 |
|  | Forewing Maximum | 0.006 | -0.087 | 0.102 | 0.44 |
|  | Voltinism | -0.135 | -0.193 | -0.068 | 0.92 |

**Table S2a.** Results of Mantel tests conducted between phylogenetic distance and abundance asynchrony for each taxon, along with 95% confidence intervals.

| Taxa | Mantel R | P Value | Lower CI | Upper CI |
| --- | --- | --- | --- | --- |
| Lepidoptera | 0.0304 | 0.01 | 0.0204 | 0.0412 |
| Macro-moths | 0.057 | 0.0001 | 0.0477 | 0.0691 |
| Butterflies | 0.137 | 0.01 | 0.0876 | 0.189 |
| Bumblebees | -0.0503 | 0.702 | -0.144 | 0.0305 |
