# Supplementary material for: Asynchrony in terrestrial insect abundance corresponds with species traits
Source: Ecol Evol. 2024 Jan 31;14(2):e10910. doi: 10.1002/ece3.10910 (PMC10830349; doi:10.1002/ece3.10910)
Supplement: Supplementary file 3 — Appendix S3. [file ECE3-14-e10910-s003.docx]

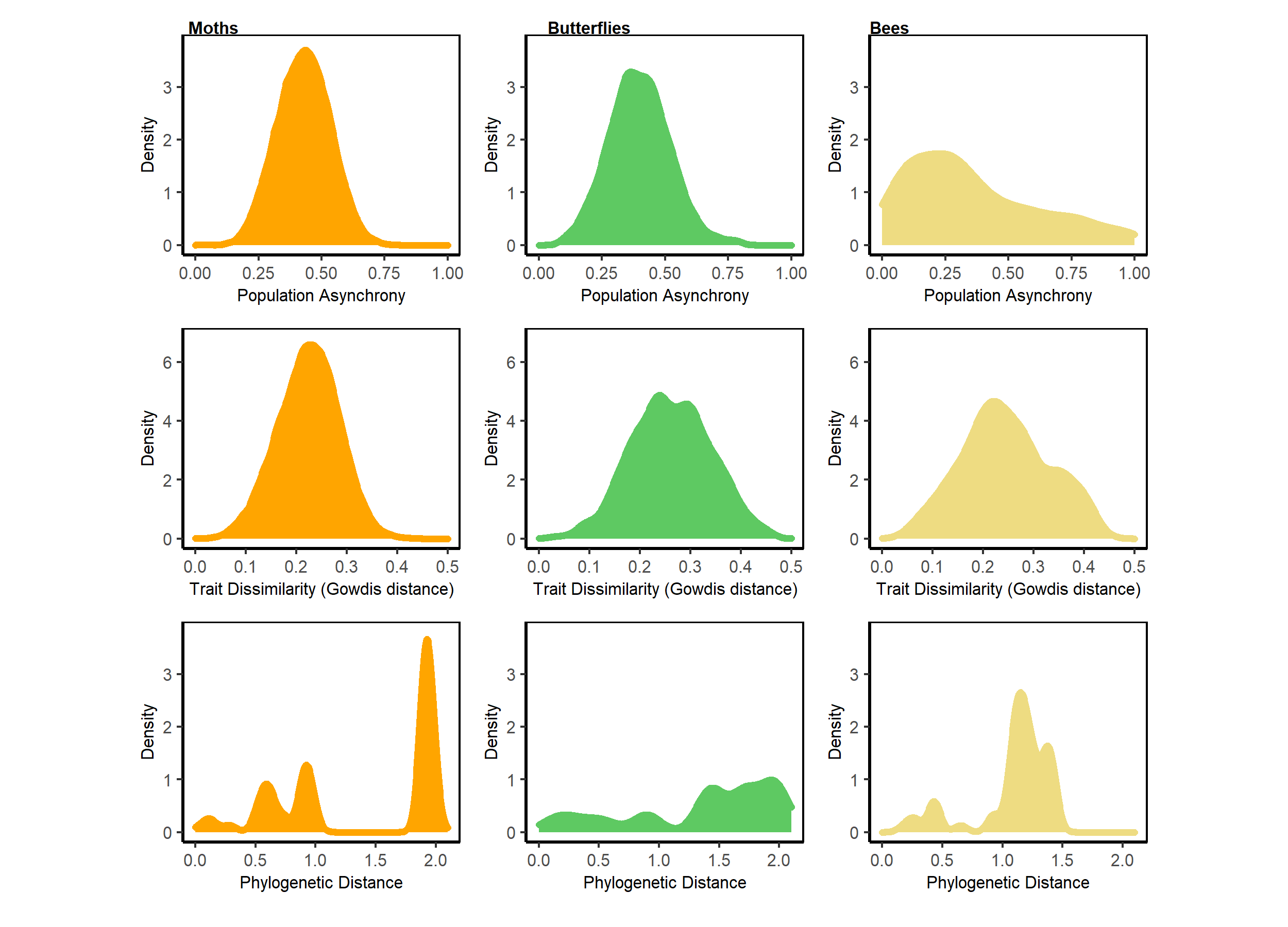
Appendix S3

**Fig S3.** Distribution of pairwise interspecific dissimilarity values produced for matrices for interannual population changes (top), functional traits (middle), and phylogeny (bottom) across macro-moths (left), butterflies (middle) and bumblebees (right).
